# A 5-Year Review of Senecavirus A in China since Its Emergence in 2015

**DOI:** 10.3389/fvets.2020.567792

**Published:** 2020-09-30

**Authors:** Fuxiao Liu, Qianqian Wang, Yilan Huang, Ning Wang, Hu Shan

**Affiliations:** College of Veterinary Medicine, Qingdao Agricultural University, Qingdao, China

**Keywords:** senecavirus A, China, epidemiology, phylogenetic analysis, diagnostics, vaccines

## Abstract

Senecavirus A (SVA), previously known as Seneca Valley virus, is classified into the genus *Senecavirus* in the family *Picornaviridae*. This virus can cause vesicular disease and epidemic transient neonatal losses in swine. Typical clinical signs include vesicular and/or ulcerative lesions on the snout, oral mucosa, coronary bands and hooves. SVA emerged in Guangdong Province of China in 2015, and thereafter gradually spread into other provinces, autonomous regions and municipalities (P.A.M.s). Nowadays more than half of the P.A.M.s have been affected by SVA, and asymptomatic infection has occurred in some areas. The phylogenetic analysis shows that China isolates are clustered into five genetic branches, implying a fast evolutionary speed since SVA emergence in 2015. This review presented current knowledge concerning SVA infection in China, including its history, epidemiology, evolutionary characteristics, diagnostics and vaccines.

## Introduction

Senecavirus A (SVA), also known as Seneca Valley virus (SVV), belongs to the genus *Senecavirus* in the family *Picornaviridae*. SVA now is the only member in the genus *Senecavirus*, and has only one serotype (1). This virus was initially identified incidentally as a contaminant in cell culture medium during cultivation of PER.C6 (transformed fetal retinoblast) cells. SVA is not pathogenic to normal human cells, but has potent oncolytic activity in tumor cells, such as neuroendocrine tumor cells (2) and human retinoblastoma cells (3).

SVA can cause vesicular disease and epidemic transient neonatal losses (ETNL) in swine. In 2007, dozens of pigs at a Canada market showed idiopathic vesicular disease (IVD)-like clinical signs: broken vesicles along the coronary band that was swollen and blanched white, with tissues separating from the edge of hoof and dewclaws sloughing from their attachments. The IVD was subsequently diagnosed with SVA infection (4). In 2010, a 6-month-old intact male Chester White boar exhibited the IVD-like clinical signs in the USA, and then was diagnosed with SVA infection (5). At the end of 2014 and the beginning of 2015, frequent outbreaks of SVA infection were reported in weaned and adult pigs in different geographical regions of Brazil (6–9).

In 2015, vesicular lesions were observed in pigs at farms in Guangdong Province of China, where newborn piglets presented with sudden death at the same time. This case was diagnosed with SVA infection, the first outbreak in China (10). In the following years, SVA gradually spread into other provinces. By December 2019, more than half of the provinces, autonomous regions and municipalities (P.A.M.s) had been reported to be affected by SVA infection in China. The phylogenetic analysis shows that China isolates can be mainly grouped into five genetic branches (11). Events of genetic recombination have occurred among a few China strains (12–14), and asymptomatic SVA infection has also been found in some areas (15, 16). Here, we comprehensively reviewed a 5-year history (2015–2019) of SVA infection in China, including its history, epidemiology, evolutionary characteristics, diagnostics, and vaccines.

## SVA Characteristics

### Viral Structure

**Figure 1:**
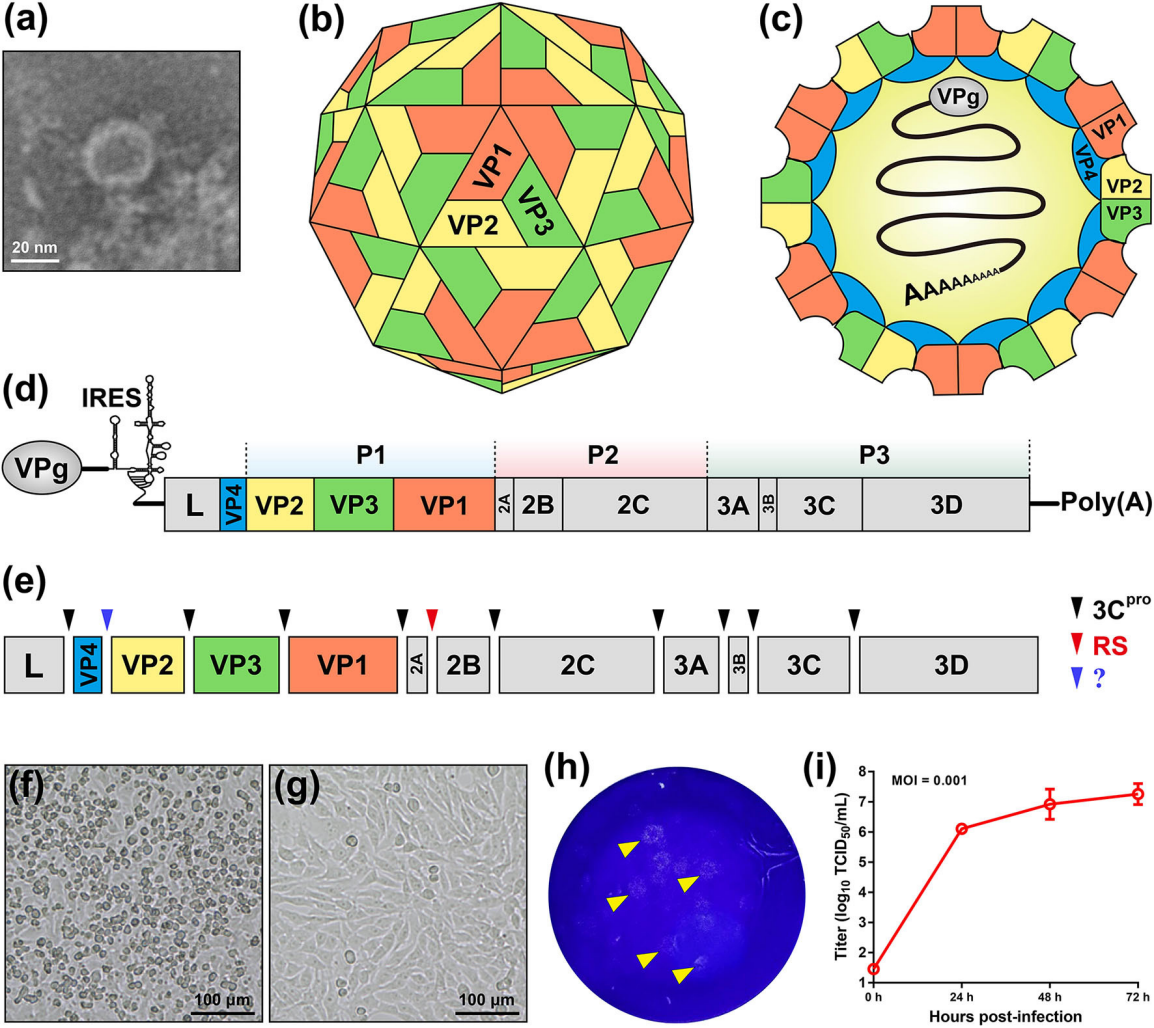
Characteristics of Senecavirus A. Transmission electron microscopy of purified SVA virion **(a)**. Schematic representations of SVA virion: front **(b)** and section **(c)** views. Schematic representation of SVA genome **(d)**. Cleavage sites (arrow-headed) within SVA polyprotein **(e)**. Typical cytopathic effect, cell rounding, at 24 h post infection with SVA **(f)**, and non-infected cell monolayer as mock **(g)**. Plaque formation (arrow-headed) of SVA-infected cell monolayer **(h)**. Multi-step growth curve (MOI = 0.001) of rescued SVA CH-LX-01-2016 cultivated in BSR-T7/5 cells **(i)**. IRES, internal ribosome entry site; 3C^pro^, 3C protease; RS, ribosomal skipping; ?, unknown.

SVA is the only member in the genus *Senecavirus*, and shares many features with other picornaviruses. Morphologically, the mature virion of SVA is a non-enveloped icosahedral (T = pseudo3) particle with a diameter of ~27 nm. Purified virion or, less probably, procapsid (17), reveals a sphere-like shape under our observation by transmission electron microscopy (Figure 1a). The viral capsid is composed of a densely-packed icosahedral arrangement of 60 protomers, each consisting of four structural proteins (VP1, VP2, VP3, and VP4). The VP1, VP2, and VP3 are “spliced” together to form an outer shell (Figure 1b); and the VP4 is located beneath the internal side of the capsid (Figure 1c). A viral protein (VPg) is covalently linked to the 5′ end of viral RNA genome, encapsidated inside the virion (Figure 1c).

### Viral Genome

The SVA genome is a positive-sense, single-stranded, non-segmented, and linear RNA, ~7.3 nt in length, with a 3′ poly (A) tail but without a 5′ capped structure (Figure 1d). The genome contains 5′ and 3′ untranslated regions (UTR), and a single long open reading frame (ORF) of polyprotein precursor. The 5′ UTR contains a sequence of type IV internal ribosome entry site (IRES) (18), which has structural and functional similarities to those of pestiviruses (Figure 1d) (19), allowing for translation initiation in a cap-independent manner. The 3′ UTR is ~70 nt in length, and reveals a kissing-loop structure, followed by a poly (A) sequence. The polyprotein ORF consists of the leader (L) and 3 major protein regions (P1, P2, and P3), and follows the standard “L−4–3–4” layout, namely “Leader−4 polypeptides of P1–3 polypeptides of P2–4 polypeptides of P3” or “L–VP4–VP2–VP3–VP1–2A−2B−2C−3A−3B−3C−3D”. The first SVA isolate from China (20), CH-01-2015 (Genbank access No.: KT321458), has an ~7,300-nt-long genome, which contains a 668 nt 5′ UTR, a 64 nt 3′ UTR and a 6,543 nt ORF that encodes a 2,181 aa polyprotein.

### Viral Proteins

Referring to other picornaviruses (21), a polyprotein precursor of SVA can be stepwisely cleaved into twelve polypeptides (Figure 1e). The primary “cleavage” event is involved in a mechanism of ribosomal skipping, and occurs at the conserved TNPG↓P motif between the 2A and the 2B, in which the arrow indicates the “cleavage” site. The 2A performs the function of ribosomal skipping. In the P1 region, the P1 polypeptide is cleaved into VP0, VP3, and VP1 by the 3C protease. The precursor VP0 will be cleaved further into VP4 and VP2, and however the cleavage mechanism remains unclear. The VP1, VP2, VP3, and VP4 are structural proteins, required for encapsidation of SVA genome. The P2–P3 region is cleaved into seven non-structural proteins: 2A, 2B, 2C, 3A, 3B (VPg), 3C (protease), and 3D (polymerase). The 3B is the genome-linked polypeptide, sharing a low homology with those of other picornaviruses, and functions as a protein primer for RNA synthesis. The 3C is a protease, responsible for cleaving the SVA polyprotein precursor. The 3D is a RNA-dependent RNA polymerase, functioning in virus replication and VPg uridylylation (22).

### Growth Characteristics *in vitro*

Many cell lines, such as BHK-21 (baby hamster kidney-21), PK-15 (porcine kidney-15) and ST (swine testis), can be used for isolation and cultivation of SVA (23). SVA-inoculated cells would exhibit typical cytopathic effects (CPE), like cell rounding and detachment (Figure 1f), compared with a non-infected cell monolayer (Figure 1g). Plaque morphologies generally depend on SVA isolates and cell lines. Figure 1h shows SVA-induced plaque formation on a BSR-T7/5 cell monolayer at 72 h post infection (hpi). SVA replication is able to reach a high level of peak titer in its susceptible cells. Wang et al. (24) recently compared growth kinetics of five SVA isolates from Guangdong Province of China. All of them displayed similar growth kinetics to one another in the ST-R cell line, and could reach their peak titers (10^8^-10^9^ TCID_50_/mL) at 48 hpi (11). We recently rescued a China strain (CH-LX-01-2016) using reverse genetics, and its growth kinetics was measured during 0 and 72 hpi in BSR-T7/5 cells (Figure 1i). It subsequently underwent serial 80 passages *in vitro* for genetic analysis by next-generation sequencing, which revealed neither sequence-deleting nor -inserting phenotype detectable in viral progenies, suggesting a relatively high stability of viral nucleic acids during consecutive passages.

### Viral Pathogenesis

**Figure 2:**
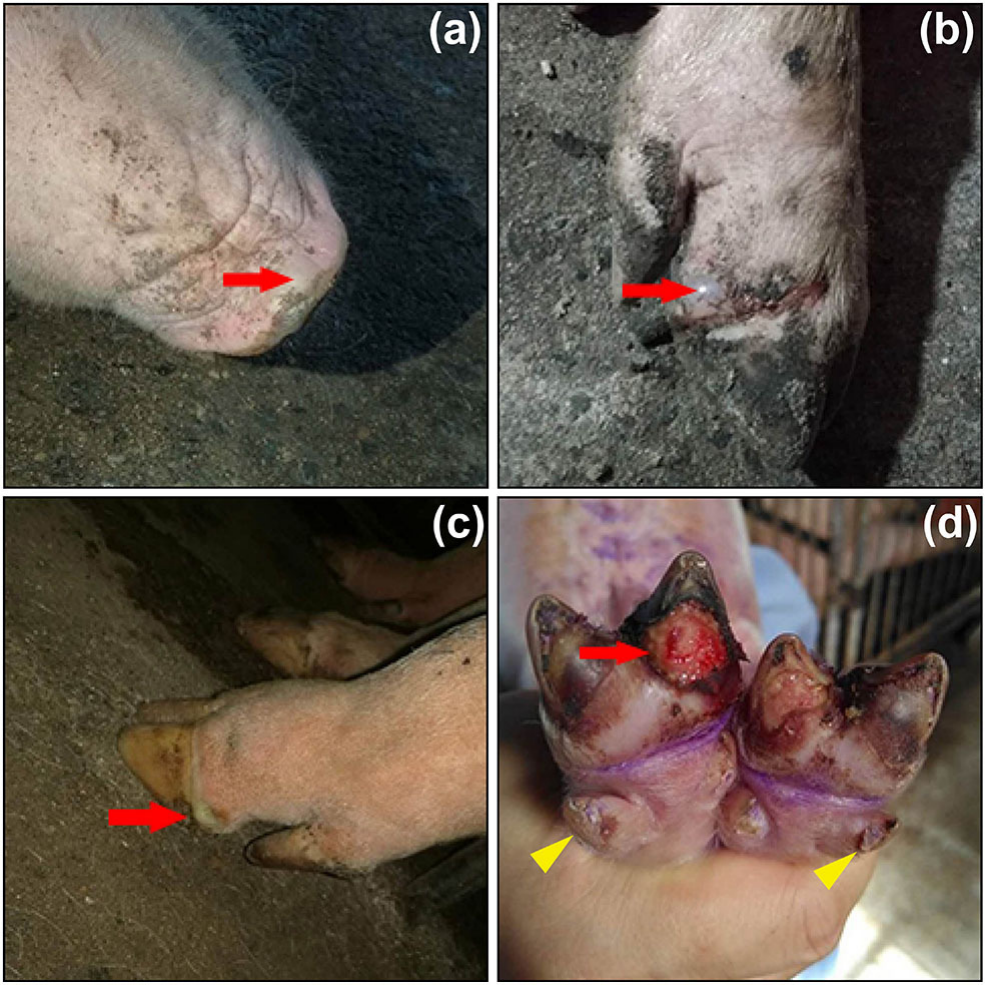
Clinical signs of pigs infected with Senecavirus A in Fujian Province of China in 2017. Fluid-filled vesicles on snout (**a**, red arrow) and coronary bands (**b,c**, red arrows). Ruptured vesicle with ulceration and erosion beneath the toe (**d**, red arrow), and lesions on dewclaws (**d**, yellow arrowheads). Adapted from the reference (23) with permissions of the Elsevier (License No.: 4892830654384).

Clinical signs of SVA infection are indistinguishable from those of other vesicular diseases, caused by foot-and-mouth disease virus (FMDV), swine vesicular disease virus, vesicular stomatitis virus and vesicular exanthema of swine virus (1). Typical clinical signs are vesicular and/or ulcerative lesions on the snout, oral mucosa, coronary bands and hooves (Figure 2). Rupture of a fluid-filled vesicle would leave erosion on the skin, subsequently evolving to form a crust on the affected area. Some herds additionally suffer an increase in neonatal losses of 1- to 4-day-old piglets, termed ETNL (25). Other clinical signs include lethargy, lameness, anorexia and even diarrhea.

Experimental infection of SVA in finishing pigs revealed that tonsil was one of the primary sites of SVA replication during the acute stage of infection. The viremia period was relatively short, lasting ~7 days, whereas virus shedding was detected between 1 and 28 days post infection in oral and nasal secretions and feces. Some historical strains, albeit detected in pigs presenting with vesicular disease, failed to reproduce the disease in virus-inoculated pigs (26).

## Emergence, Spread, and Influence of SVA in China

### First Outbreak in 2015

In March 2015, an outbreak of vesicular disease occurred in Guangdong Province of China. The clinical signs were mainly characterized by vesicular lesions in sows and acute death of neonatal piglets. Conventional differential diagnoses were carried out to exclude foot-and-mouth disease, swine vesicular disease, vesicular exanthema of swine and vesicular stomatitis. Wu et al. (10) designed seven pairs of primers for amplifying the complete genome of SVA by RT-PCR assays. Sequence alignment showed that the emerging virus shared 94.4–97.1% nucleotide identities with eight SVA strains (10). This was the initial report of SVA infection in China. The first China isolate was the CH-01-2015 (20), clustered in the same clade as that of the USA strains. The earliest isolates from Guangdong Province, including the CH-01-2015, CH-02-2015, CH-04-2015, CH-DB-11-2015, CH-LX-01-2016, CH-DL-01-2016, and CH-ZW-01-2016, were clustered into two different clades by a phylogenetic analysis. At the nucleotide level, they shared 96.3–97.1 and 97.9–98.3% similarities with the USA and Brazil strains, respectively (27).

### SVA Spread since 2015

Since its emergence in Guangdong, the SVA continuously spread into other P.A.M.s. The second SVA-affected province was Hubei in March 2016, when an outbreak of vesicular disease occurred in piglets at a pig farm (28). An infectious SVA was isolated and named HB-CH-2016 (Genbank access No.: KX377924), which shared an extremely high (99%) homology of genome with the CH-01-2015, implying a significant epidemiological correlation between the first two events of SVA outbreak in China. In December 2016, finishing pigs exhibited typical signs of vesicular disease at a farm of Heilongjiang Province in northeast China. A SVA strain was isolated and designated SVA/HLJ/CHA/2016 (Genbank access No.: KY419132) (29). Its genome shared high nucleotide identities (93.8–99%) with others deposited in Genbank. Interestingly, it was genetically closer to the 2015 USA strains than to those reported earlier in China.

In January 2017, SVA emergence was almost simultaneously found in two non-neighboring provinces, Fujian and Henan (30). Three strains, CH-FJ-2017 (Genbank access No.: KY747510), CH-HN-2017 (Genbank access No.: KY747511) and CH-HNSL-2017 (Genbank access No.: KY747512) were subsequently isolated for phylogenetic analysis. The nucleotide sequence identities ranged from 99.7–99.8% among the three isolates. They shared the highest homologies of genome (98.8–98.9%) and polyprotein (99.5–99.6%) with the KS15-01 strain, rather than with the earlier China isolates. Meanwhile, other two strains, CH/FuJ/2017 (23) and CH-HNKZ-2017 (31), were isolated from Fujian and Henan Provinces, respectively. Both isolates were proven to share the highest genetic homologies with two USA isolates, USA/GBI29/2015 and US-15-40381IA, respectively. In the same year, SVA infection was identified at a pig farm in Gansu Province (32), which was not adjacent to other provinces affected by SVA earlier. A phylogenetic analysis showed that the Gansu isolate CH-LZ-2017 shared the highest nucleotide homology (99%) of VP1 with that of the Henan strain CH-HN-2017.

In August 2018, SVA emerged in Guangxi, a neighboring province of Guangdong. Phylogenetic analyses indicated that the Guangxi isolate CH-GX-01-2018 shared the highest homology (98.6%) with those isolated from Guangdong in 2017 at the genome level (33), and the other Guangxi isolate CH-GX-02-2018 also revealed the highest genetic identity with several Guangdong strains (34), implying a possible epidemiological relationship between Guangxi and Guangdong.

### SVA Reemergence

It was recently reported that SVA reemerged in some provinces, especially in Guangdong. In 2017, four groups independently isolated and further analyzed reemerging strains in Guangdong. The first group isolated 17 novel strains, and revealed their complete genomes, VP1, 3C, and 3D genes sharing 96.5–99.8, 95.1–99.9, 95.6–100, and 96.9–99.7% nucleotide identities with one another, respectively (35). The second group isolated six strains, and revealed nucleotide identities among their genomes ranging from 99.6 to 99.8%. These six strains shared the highest genomic identity with the USA/GBI29/2015 (98.5–98.6%), and were notably distinct from earlier isolates from China (36).

The third group determined two strains, CH-GD-2017-1 and CH-GD-2017-2, both of which showed different characteristics from those of earlier isolates in Guangdong, but were more similar to the USA strains (37). The forth group isolated five strains, GD01/2017, GD03/2017, GD04/2017, GD05/2017, and GD06/2017, subsequently subjected to genomic sequencing. The result revealed that GD01/2017 and GD03/2017 shared an extremely high homology with each other (99.4%), were both closely related to the USA/GBI29/2015 (98.6%), but showed relatively low homologies with GD04/2017, GD05/2017, and GD06/2017. All five isolates were classified into four different genetic clades by a phylogenetic analysis, suggesting a low genetic homology among them, except that between GD01/2017 and GD03/2017. Such a great diversity among their genomes implied that local strains in Guangdong had evolved into different branches (11).

### Current Status of SVA Infection

In addition to Guangdong, Hubei, Heilongjiang, Fujian and Henan, other P.A.M.s have actually been affected by SVA. During 2016 and 2018, the China Animal Health and Epidemiology Center (CAHEC) collected hundreds of samples from 15 P.A.M.s, including Hunan, Yunnan, Xinjiang, Liaoning, Fujian, Hubei, Guangxi, Hebei, Jiangxi, Jilin, Sichuan, Shandong, Shannxi, Shanghai, and Guizhou. More recently, the CAHEC reported the result of detection for all samples, showing that, out of these 15 P.A.M.s, only four provinces, Hebei, Jiangxi, Jilin, and Shannxi, were not affected by SVA infection (38). As to Jilin, a recent report, nonetheless, revealed that pigs actually had been subclinically infected by SVA at a few farms in this province (39).

**Figure 3:**
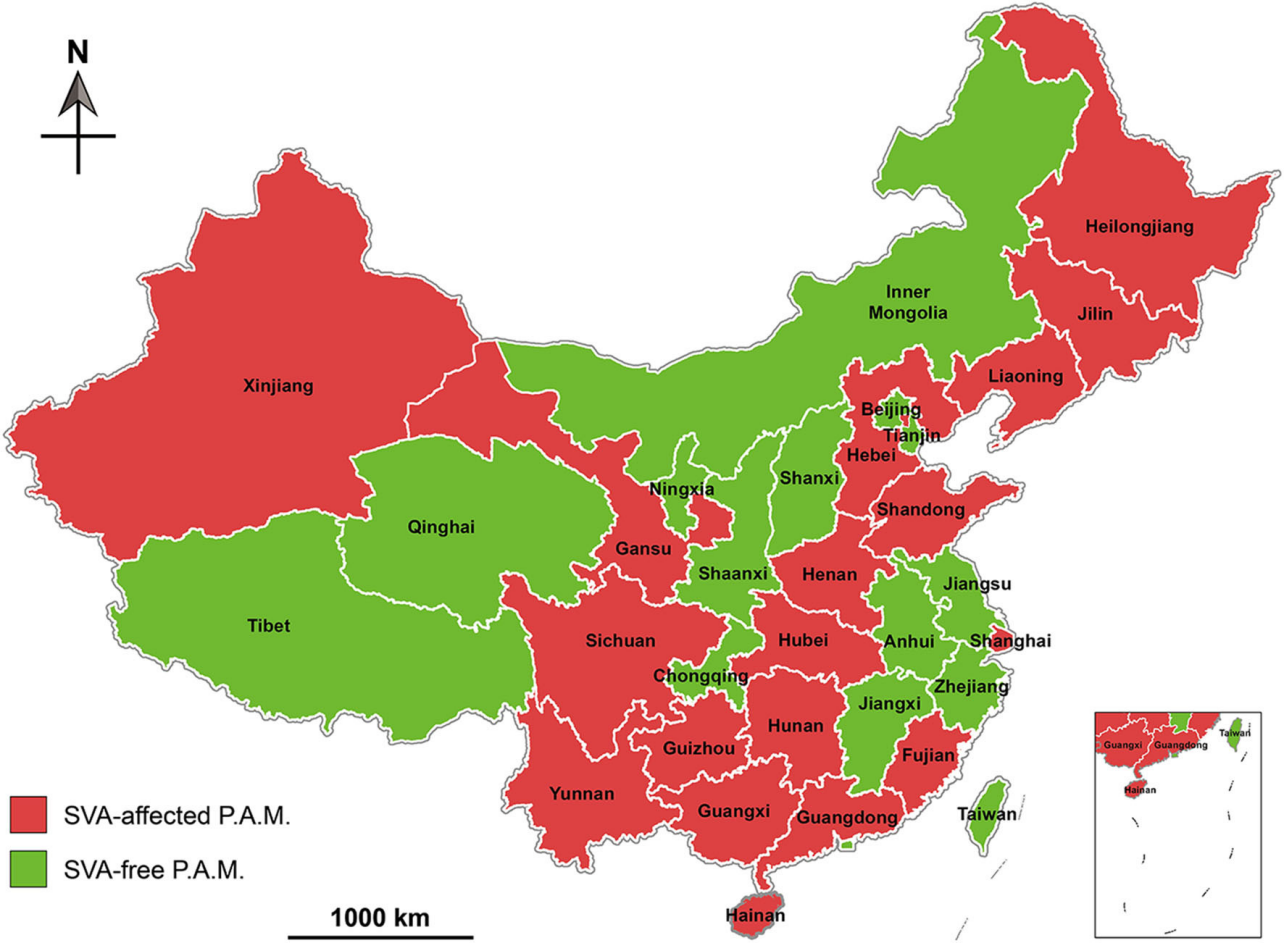
Geographical distribution of Senecavirus A-affected and -free P.A.M.s in China during 2015 and 2019. P.A.M, province, autonomous region and municipality.

Unlike other P.A.M.s, Hainan Province is an ocean island, and therefore not prone to be affected by animal diseases. A report, nevertheless, revealed that out of 2,547 serum samples collected from Hainan in 2018, 278 were diagnosed SVA antibody-positive by ELISA analysis (40). Based on existing reports, we created a map that exhibited geographical distribution of SVA-affected P.A.M.s in China during 2015 and 2019 (Figure 3), and key data were listed in Table 1.

**Table 1:** SVA-affected provinces, autonomous regions and municipality in China during 2015 and 2019.

**P.A.M**.	**Year of SVA emergence**	**Representative isolate**	**Genbank access No**.	**References**
Guangdong	2015	CH-01-2015	KT321458	(20)
Hubei	2016	HB-CH-2016	KX377924	(28)
Heilongjiang	2016	SVA/HLJ/CHA/2016	KY419132	(29)
Fujian	2017	CH-FJ-2017	KY747510	(30)
Henan	2017	CH-HN-2017	KY747511	(30)
Gansu	2017	CH-LZ-2017	NA	(32)
Guangxi	2018	SVA/GX/CH/2018	MK039162	(33)
Shandong	NA	SVV-CH-SD	MH779611	(41)
Hainan	2018	NA	NA	(40)
Xinjiang	NA	NA	NA	(38)
Liaoning	NA	NA	NA	(38)
Hunan	NA	NA	NA	(38)
Yunnan	NA	NA	NA	(38)
Sichuan	NA	NA	NA	(38)
Shanghai	NA	NA	NA	(38)
Guizhou	NA	NA	NA	(38)
Jilin	NA	NA	NA	(39)

*P.A.M., province, autonomous region and municipality; NA, not available*.

### Phylogenetic Analysis of SVAs

**Figure 4:**
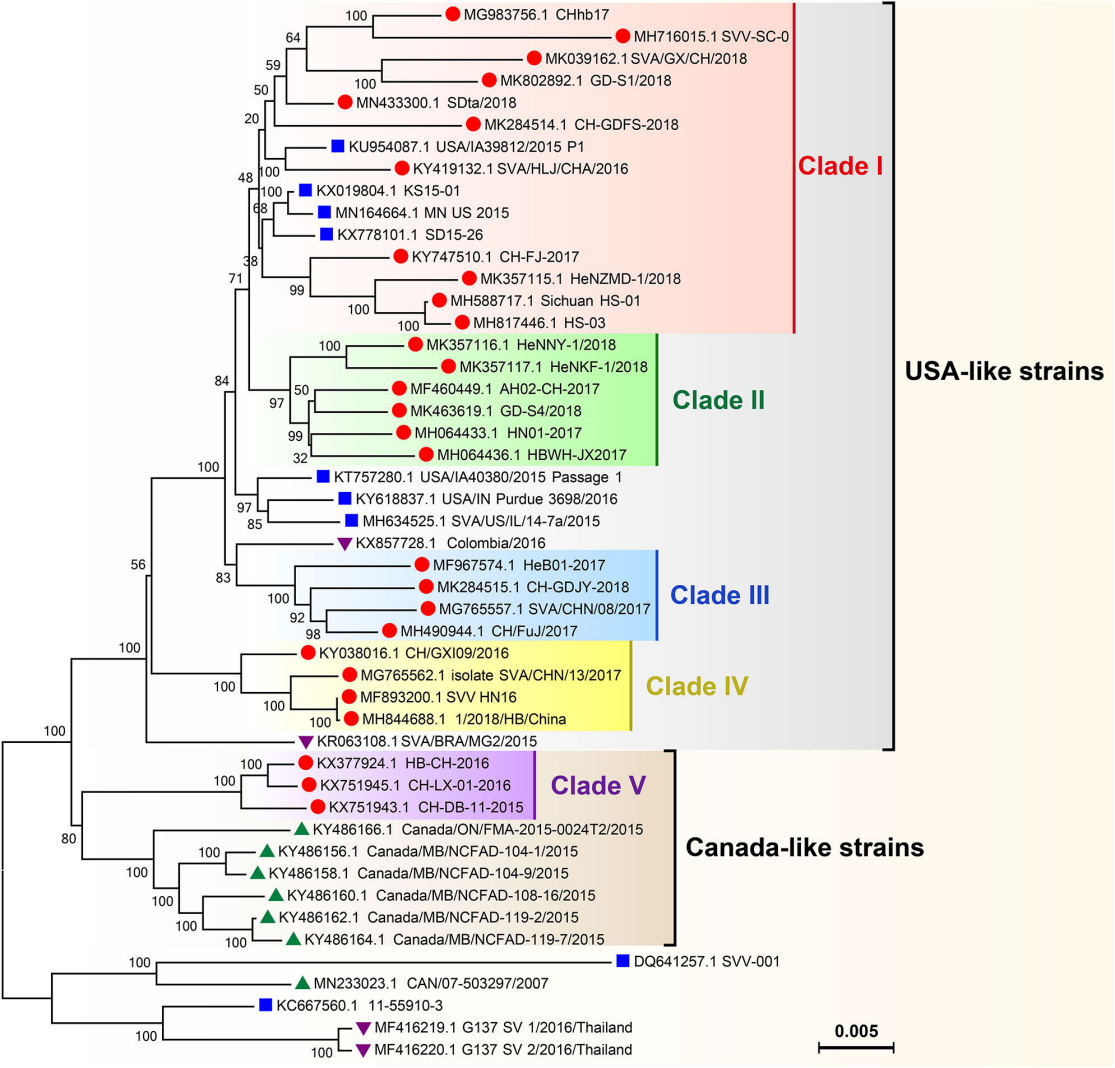
Phylogenetic analysis on genomes of SVA isolates. The Neighbor-joining method and Kimura-2-parameter model were used for construction of the phylogenetic tree using MEGA 7.0 software. Numbers indicated the bootstrap value (1,000 replicates). SVA isolates from China, the USA, Canada and other countries were marked with “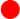,” “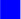,” “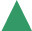,” and “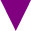,” respectively.

Phylogenetic analysis of global SVA genomes revealed two major evolutionary clusters, USA- and Canada-like strain clusters. To date, more than half of the P.A.M.s have been affected by SVA in China (Figure 3). Almost all isolates from these P.A.M.s are classified into both clusters. Additionally, all China isolates could be mainly grouped into five genetic branches, namely clade I, II, III, IV, and V (11). Here, we constructed a phylogenetic tree (Figure 4), by which a part of China isolates were compared with exotic ones for showing their evolutionary relationship. The phylogenetic analysis reveals that clade I, II, III, and IV belong to the USA-like cluster, and only the clade V belongs to the Canada-like cluster, suggesting that the USA-like strains are predominant in China. A total of five clades in Figure 4 demonstrate that the current genetic relationship become more complex than the past one among China isolates. SVA has been evolving in China since its emergence in 2015.

### Recombination Events Between SVAs

SVA is one of RNA viruses, characterized by relatively high rate of genetic mutation during their replication *in vitro* and *in vivo* (42). Genomic recombination by a copy-choice mechanism is a genetic feature of picornaviruses (43), and unfortunately is a widely neglected issue during evolutionary analysis of variants. In addition, picornaviruses generally have a poor ability to accommodate stably foreign genes. Using reverse genetics, we previously rescued two recombinant SVAs, both of which separately expressed a green fluorescent protein (44) and a luciferase. It has been demonstrated that both recombinant SVAs are genetically unstable and prone to losing their foreign sequences during serial passages *in vitro*.

SVA has been continually evolving, and even recombining with one another in field since its emergence in China. A SimPlot analysis revealed two breakpoints that separated the genome of China SVA isolate HeN-1/2018 into three regions, out of which two (1–959 and 2,355–7,312 nt) derived from the USA/IA44952/2015, and the other (960–2,354 nt) derived from the USA/IN_Purdue_4885/2015 (14). The SimPlot analysis implied a possible even of recombination between both USA strains (or their progenies) to form the emerging China strain. Liu et al. (12) isolated two novel SVAs, CH-GDFS-2018 and CH-GDJY-2018, from slaughtered pigs, which had recovered from the vesicular disease. Their recombinant fragments comprised both structural and non-structural protein regions, involved in genetic recombination between local (or domestic) and foreign strains (12).

More recently, Guo et al. (13) reported that one isolate (HeNNY-1/2018) and two previously reported strains (HB-CH-2016 and SVA/CHN/10/2017) were identified as recombinants using several algorithms. It revealed that the recombination among SVA strains had occurred in China since 2016 or earlier (13). The frequent recombination events caused emergence of novel variants, consequently increasing the complexity of SVA spread in China. Considering the adaptation of SVA to pigs, the occurrence of natural recombination is of clinical importance.

### Pathogenesis of China Isolates

Four groups independently reported experimental infection of SVA China isolates in animals. The first group inoculated pigs with two SVA isolates, GD-S5/2018 and GD04/2017. The result showed that the GD-S5/2018 was a virulent isolate, while the GD04/2017 was nearly avirulent. Both strains shared 93.9% nucleotide identity with each other, whereas the mechanism, involved in impact of genomic variance on virulence, remained unclear (45). The second group compared other two isolates, HB-CH-2016 and CH/AH-02/2017, for evaluating their pathogenesis in pigs. The result revealed that SVA-infection-like clinical signs were observed only in CH/AH-02/2017-infected pigs (46). Both isolates shared 96% homology with each other at the genomic level, and exhibited 29 amino acid differences. The HB-CH-2016 and CH/AH-02/2017 belonged to the Canada- and USA-like strains, respectively. The USA-like strains have become predominant among China isolates since 2017.

The third group inoculated 30–35-, 55–65-, and 90–100-day-old pigs with a clinical isolate (SVV-CH-SD) from Shandong Province. The result revealed that 90–100-day-old pigs developed low-grade fever, blisters and lameness, whereas neither 30–35- nor 55–65-day-old pigs showed typical clinical signs, suggesting the SVV-CH-SD with age-dependent variation in virulence (41). The forth group isolated one SVA (SVA-FJLY strain) from Fujian Province in 2018, with which subsequently three pigs were intranasally inoculated (10^9^ TCID_50_/pig). Two SVA-infected pigs developed vesicular and ulcerative lesions on their snouts and oral mucosa, and the other had no typical clinical signs (47). The results from these four groups suggested that high- and low-virulence SVAs were simultaneously circulating in China. Indeed, subclinically infected cases are growingly identified in China.

## Diagnostics and Vaccines Developed in China

### Diagnostics

A variety of conventional and novel methods have been established for diagnosing SVA infection. Non-Chinese groups have developed two kinds of major methods, competitive ELISA (48, 49) and distinct RT-PCRs (50–56). Some of them have been used as commercially available kits for clinical detection.

Meanwhile, Chinese researchers also reported nucleic acid-based methods, characterized by high specificity and sensitivity, and having a potential in clinical detection of SVA. These methods mainly include conventional RT-PCR (57), quantitative RT-PCR (qRT-PCR) (24, 58), RT-droplet digital PCR (RT-ddPCR) (59), reverse transcription loop-mediated isothermal amplification (RT-LAMP) (60), RT-LAMP-lateral flow dipstick (RT-LAMP-LFD) (61), recombinase polymerase amplification (RPA) and RPA-LFD (62). Their detailed data are listed in Table 2.

**Table 2:** Nucleic acid-based diagnostics of SVA infection developed in China.

**Diagnostics**	**Target sequence**	**Limit of detection**	**No cross-reaction with**	**References**
RT-PCR	5′ UTR	1 TCID_50_/mL	EMCV, FMDV, PRV, PRRSV, CSFV, and PEDV	(57)
qRT-PCR	VP1	10 copy/μL	VSV, APPV, PRV, and FMDV	(58)
qRT-PCR	3D	10.4 copy/μL	FMDV, PRRSV, PEDV, PCV2, and PRV	(24)
RPA	3D	28 copy/μL	FMDV, CSFV, PRRSV, PEDV, and TGEV	(63)
RT-LAMP	VP1 and VP2	1 TCID_50_/mL	PCV2, PEDV, CSFV, PRRSV, PRV, VSV, SVDV, and FMDV	(60)
RT-ddPCR	3D	1.53 ± 0.22 copy/reaction	FMDV, VSV, SVDV, PRRSV, CSFV, and PCV2	(59)
RT-LAMP-LFD	3D	11 copy/μL	FMDV, SVDV, VSV, PDCoV, PEDV, TGEV, PRRSV and CSFV	(61)
RPA-LFD	3D	56 copy/μL	FMDV, CSFV, PRRSV, PEDV, TGEV, PRV, and PCV	(62)

*qRT-PCR, quantitative RT-PCR; RPA, recombinase polymerase amplification; RT-LAMP, reverse transcription loop-mediated isothermal amplification; RT-ddPCR, RT-droplet digital PCR; RT-LAMP-LFD, reverse transcription loop-mediated isothermal amplification-lateral flow dipstick; RPA-LFD, recombinase polymerase amplification-lateral flow dipstick; EMCV, encephalomyocarditis virus; FMDV, foot-and-mouth disease virus; PRV, pseudorabies virus; PRRSV, porcine reproductive and respiratory syndrome virus; CSFV, classical swine fever virus; PEDV, porcine epidemic diarrhea virus; VSV, vesicular stomatitis virus; APPV, atypical porcine pestivirus; PCV2, porcine circovirus 2; TGEV, transmissible gastroenteritis virus; SVDV, swine vesicular disease virus; PDCoV, porcine deltacoronavirus*.

Of all the above-mentioned methods, the qRT-PCR is now most broadly used as a nucleotide-based method for clinical detection. The novel RT-ddPCR is a third-generation PCR technique, and its detection limit was 1.53 ± 0.22 copies of SVA RNA/reaction, ~10-fold greater sensitivity than that of the qRT-PCR (59). RPA is another novel tool, a highly sensitive and selective isothermal amplification technique for detecting viral nucleic acids. The lowest concentration of 28 copies of SVA RNA/μL could be detected within 10 min at 40°C (63). These methods have their own relative merits, can complement but cannot substitute for one another.

Besides the nucleotide-based methods, serological diagnostics also plays a key role in clinical detection of SVA. The joint use of both methods would enhance the accuracy of diagnosis. Zhao et al. (64) developed a VP1-based indirect ELISA, which could be used for detection of SVA-induced antisera, and conferred no cross-reaction with those of common swine viruses (64). In addition to the VP1, the VP2 could also be used for establishment of serum diagnostics. The VP2-based block and indirect ELISAs showed high sensitivity and specificity for anti-SVA sera (65, 66). Using the recombinant SVA that expressed a green fluorescent protein, we recently developed a rapid virus neutralization test (VNT) for detecting serum samples. The novel VNT shortened the period of 96-well plate incubation to 48 h, much less than that of the conventional VNT. Moreover, it was more sensitive and specific than the conventional VNT using wide-type SVA, because a final VNT result of readout would depend on green fluorescence, rather than CPE (44).

### Vaccines

Compared with the numerous studies of diagnostics, those of SVA vaccines have been rarely reported as yet. Despite no commercially available vaccines now, the unique SVA serotype makes the applicable scope of a given candidate broader than those of multi-serotype viruses. Moreover, the SVA can efficiently propagate in BHK-21 cells to reach a high titer, even more than 10^9^ TCID_50_/mL in culture supernatant, greatly facilitating mass production of seed stocks. Sharma et al. (67) developed a novel candidate of live-attenuated vaccine, with which a single immunization could confer protection against heterologous SVA challenge, as demonstrated by absence of overt disease and reduced viremia, virus shedding and viral load in tissues (67). This candidate represents a promising alternative to prevent SVA infection in pigs.

Besides the live-attenuated candidate, inactivated and DNA-based vaccines also showed the potential in stimulating immune responses. A Chinese group developed an inactivated vaccine candidate using the isolate CH-FJ-2017. The seed virus was produced in BHK-21 cells by roller bottles, inactivated with binary ethylenimine, and emulsified in oil adjuvant. The immunogenicity of inactivated SVA was evaluated in pigs by VNT. The vaccinated pigs (2 μg/dose) could develop high-titer neutralizing antibodies, and showed no clinical signs when challenged with the homologous virus, indicating a high protective efficacy of the produced vaccine (68). More recently, this group constructed a recombinant plasmid, which not only efficiently expressed a fusion polypeptide, P12A-3C, in 293T cells, but also could induce high-titer SVA-specific antibodies in mice (69). FMDV virus-like particles (VLPs) were demonstrated to have the ability to induce robust immune responses *in vivo* (70–72), which would offer a new perspective on development of novel VLP vaccines against SVA (73).

Nowadays, there are two major challenges for the development of SVA vaccines. One is screening of standard strains with high virulence for challenge tests in pigs. Clinical signs cannot possibly be reproduced after experimental infection with some low-virulence SVAs, or even with those clinically isolated from diseased pigs. We found that a few strains, albeit isolated from vesicular fluid of diseased pigs, could not cause observable clinical signs, even obvious onset of pyrexia, in experimental pigs (data not shown). The other challenge is establishment of laboratory animal models susceptible to SVA infection. Laboratory animal models have provided valuable insight into the pathogenesis and immunogenicity of FMDV in target species (74), but are still unavailable to SVA infection. We found that neither Balb/c nor Kunming mice showed obvious signs after intramuscular (7 × 10^6^ TCID_50_/mouse), subcutaneous (2 × 10^7^ TCID_50_/mouse) or intraperitoneal (2 × 10^7^ TCID_50_/mouse) injection with the rescued SVA CH-LX-01-2016. Thus, overcoming these two challenges would greatly facilitate the development of SVA vaccines.

## Conclusions

It has been 5 years since SVA emergence in China in 2015, and nowadays more than half of the P.A.M.s have been reported to be affected by SVA infection. A few provinces, like Guangdong and Henan, have been repeatedly infected, and meanwhile SVAs in these provinces have undergone inter-genetic recombination events, consequently forming novel strains. To date, SVAs have evolved into five genetic clades in China at a fast evolutionary speed. As a result, virus progenies have conferred subclinical infection of animals in some areas (15). Unfortunately, there is still no commercially available vaccine. All of these will be great challenges for the prevention and control of SVA in China. In June 2018, the Ministry of Agriculture and Rural Affairs of China issued an official notice regarding strategies for prevention and control of SVA nationwide (75). This notice would shed light on implementation of effective countermeasures against SVA in China.

## Author Contributions

FL and HS: conceptualization. FL, QW, and YH: writing—original draft preparation. NW: formal analysis. HS: project administration. All authors contributed to the article and approved the submitted version.

## Conflict of Interest

The authors declare that the research was conducted in the absence of any commercial or financial relationships that could be construed as a potential conflict of interest.
